# The use of Canakinumab in treating resistant gouty disease in patients with limited therapeutic options: The experience of the Rheumatology Clinic of Asklipeion General Hospital of Voula, Greece

**DOI:** 10.31138/mjr.28.1.48

**Published:** 2017-03-28

**Authors:** Evangelos Theotikos, Ioannis Raftakis, Antonia Elezoglou, Christodoulos Antoniadis

**Affiliations:** Asklipeion General Hospital of Voula, Athens, Greece

**Keywords:** Gout, Canakinumab, Uric Acid, Patients

## Abstract

Gout is an autoinflammatory disease caused by monosodium urate mono hydrate crystal deposition in tissues or the supersaturation of extracellular fluids of uric acid. In this study, we are going to report the experience of the Asklepeion Voula Rheumatology Clinic, treating four patients with gouty arthritis who received Canakinumab.

The crystal deposition of gout occurs when uric acid exceeds its solubility limit (approximately 6,8 mg/dl 37°C).^1,2^ Gouty disease affects adult males mostly and post-menopausal women, with higher incidence in the fifth decade of life (men/women 7–9/11). It is rare in males <30 years old and premenopausal women.^3,4^

The clinical spectrum of gout includes asymptomatic hyperuricemia, acute intermittent gout, and chronic tophaceous gout. Clinical associations include renal disease, hypertension, obesity and hyperlipidemia.^5^ The diagnosis of gout is based on the patient’s medical history, clinical examination, radiographic imaging (mostly in chronic situations), with the presence of tophi, bony erosions, soft tissue swelling, ultrasound (double contour sign) and arthrocentesis (inflammatory synovial fluid with MSU crystals on compensated polarized microscopy).^6^

Differential diagnosis of gout includes septic arthritis, pseudogout, traumatic arthritis, rheumatoid arthritis (with polyarticular presentation), seronegative arthritis.^6^

The treatment of gout can be non-pharmacological (special diet and changes in lifestyle, dealing with primary causes, avoiding specific drugs, treating comorbidities) and pharmacological. Pharmacological therapy of acute gouty arthritis includes NSAIDS, glucocorticosteroids and Colchicine. On failure of first line medication or prohibition of use due to comorbidities, second line medication can be used such as Anakinra and Canakinumab.^5,7,8,9,10^

Canakinumab is a fully human anti-IL-1b monoclonal antibody with isotype IgG1/k. It binds with great affinity specifically to human IL-1b and neutralizes its biological activity excluding its interaction with IL-1 receptors preventing gene activation and production of inflammatory mediators.^2,7,9,10^ It is indicated for the symptomatic treatment of adult patients with frequent flares of gouty arthritis (3 in the last 12 months) when NSAIDS, colchicine and the frequent use of glucocorticosteroids are contraindicated.^5,7,11,12^ The side effects studies indicated an elevated incidence for infections, but not opportunistic. Lack of reactions at the point of injection, and no increased incidence of neoplasms was observed. A small increase of uric acid levels, pancytopenia and lipid change was noted, but all were reversible.^1,6,10^

Here, we are going to report the experience of the Asklepeion Voula Rheumatology Clinic treating four patients with gouty arthritis who received Canakinumab.

The first patient, 54 years old, experienced five flares of gouty arthritis within the last year, resistant to treatment with NSAIDS, colchicine and glucocorticosteroids. At the time of his first hospitalization he had arthritis of 2^nd^ right metacarpophalangeal and 3^rd^ left proximal interphalangeal joints and both knees and ankles. Microscopic synovial fluid analysis, after arthrocentesis of the left ankle revealed MSU crystals.

He initially received colchicine and NSAIDS which were stopped due to elevation of liver enzymes. He then received per os glucocorticosteroids, 0,5 mg/kg according to the ACR 2012 guidelines, plus allopurinol. When the dose of glucocorticosteroids was lowered to 15mg of prednisone he was hospitalized for a second time with arthritis of second right metacarpophalangeal joint and olecranon bursitis. The ultrasound of 2^nd^ metacarpophalangeal joint indicated Grade 1 synovitis and the presence of tophi. He then received Canakinumab with complete symptoms recovery (concomitant use only of allopurinol).

The second patient, 32 years old, suffers from tophaceous juvenile gout from the age of 4 (no genetic test was performed due to high cost). His medical history includes at least three flares of gouty arthritis per year from a young age, nephrocalcinosis, lithotripsy at the age of 10, the use of allopurinol, colchicine, NSAIDS without response. He has multiple tophi of proximal and distal interphalangeal joints in both hands (**Figure 1**). Radiologically, he has multiple bony erosions in the carpal bones with overhanging edge sign (**Figure 2**). Ultrasound revealed double contour sign over the 2^nd^ metacarpophalangeal bone (**Figure 3**), over the knee cartilage (**Figure 4**) and a big tophus over the metatarsophalangeal joint (**Figure 5**).

**Figure 1:**
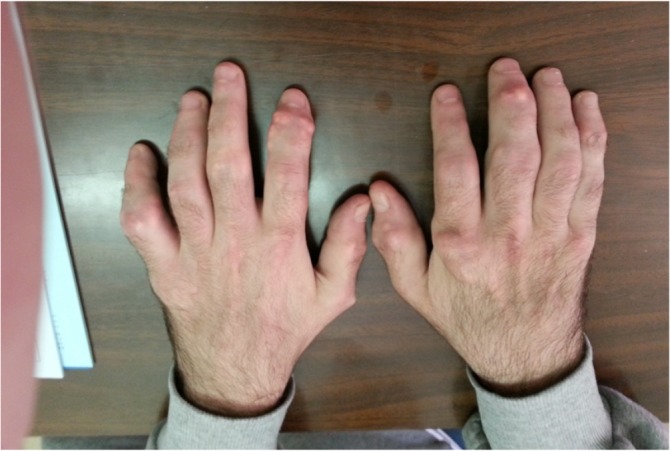
Patient 2. Multiple tophi of proximal and distal interphalangeal joints.

**Figure 2:**
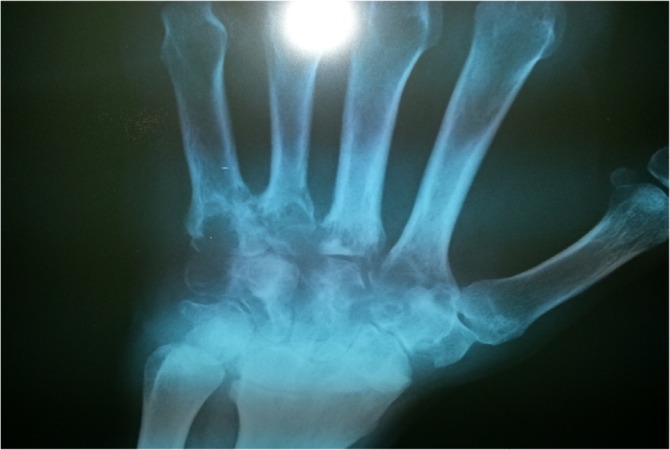
Patient 2. Multiple carpal bones erosions with overhanging edge sign.

**Figure 3:**
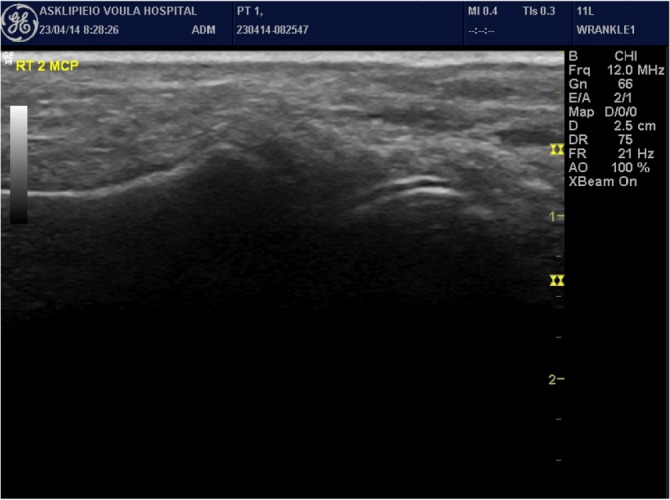
Patient 2. Ultrasound of metacarpophalangeal bone with double contour sign.

**Figure 4:**
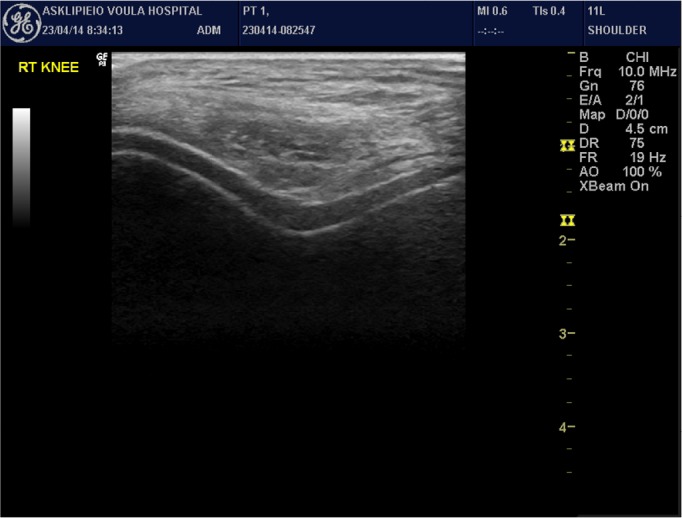
Patient 2. Ultrasound of knee cartilage with double contour sign.

**Figure 5:**
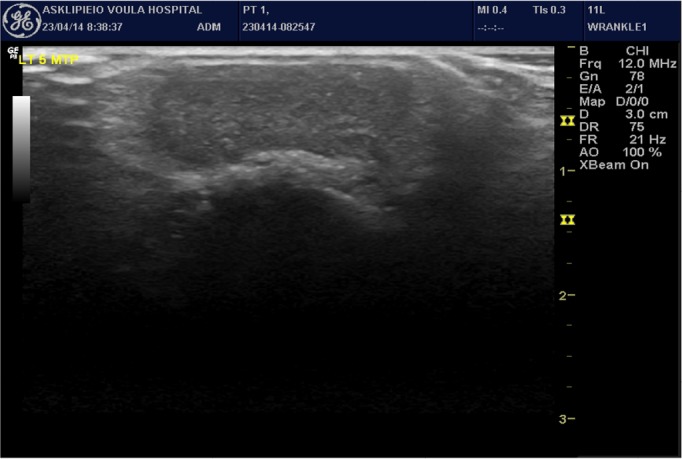
Patient 2. Metacarpophalangeal bone tophus.

The patient had progressively worsening symptoms with arthritis of both wrists, proximal interphalangeal joints, distal interphalangeal joints and both knees, initially treated with glucocorticosteroids 0,5 mg/kg according to the ACR 2012 guidelines but was non-responsive when glucocorticosteroid doses were reduced to 20 mg prednisone. He also developed intolerance to the use of colchicine and NSAIDS with abnormal liver function tests (AST 227, ALT 638, ΓGT 156) which resolved after removal. Since then, he has received 3 doses of Canakinumab with complete symptoms resolution with no flares after 3 months.

The third patient, 49 years old, has had hyperuricemia for 25 years, gout for 10 years and has multiple flares of gouty arthritis with polyarthritis of both small and large joints (metatarsophalangeals, ankles, knees, elbows). He also suffers from Type 2 insulin-dependent diabetes for 10 years and chronic kidney failure (stage 4) for 3 years. Despite the treatment with allopurinol 100 mg, prezolon 20 mg and colchicine 1/2 mg daily (adapted to chronic kidney failure) he was hospitalized 2 times with resistant polyarthritis (metatarsophalangeal joints, ankles, knees, wrists, elbows); receiving medium glucocorticosteroid doses (20–24 mg Medrol) with no improvement. No higher steroid dose was used because of difficult to control insulin dependent diabetes. His left wrist ultrasound revealed grade 3 tenosynovitis with the presence of echogenic elements within the sheath in the 4^th^ dorsal compartment (common extensor of the fingers) with possible diagnosis of gout. Ultrasound of the right knee revealed a huge joint effusion, thickened synovium with increased vascularization within the joint and the suprapatellar recess as a grade 2, power Doppler, synovitis. The double contour sign was detected over the femoral condyles cartilage suggestive of gouty disease. It also revealed chordrocalcinosis. Synovial fluid examination was positive for MSU and CPPD crystals. Because of his resistant gouty disease and the limited therapeutic options, he received Canakinumab every 3 months with complete resolution of his symptoms.

The fourth patient, 75 years old, suffers from neglected gouty disease for 20 years. He has extended presence of tophi in elbows, ankles, proximal and distal interphalangeal joints. In the last 12 months, before the initialization of Canakinumab, he had five flares of gouty arthritis localized in wrists, metacarpophalangeal and distal interphalangeal joints. He was hospitalized twice. He received treatment with NSAIDS, glucocorticosteroids (0,5 mg/kg according to the ACR 2012 guidelines), colchicine with limited response and rapid symptom reappearance after medication withdrawal. He received Canakinumab every 3 months with remission of the flares and extended free of symptoms periods.

Experience with use of Canakinumab in patients with resistant gouty disease or with comorbidities which do not allow the use of common medication seems promising so far. International studies have positive results, showing that Canakinumab 150 mg offers quick and continuing relief from pain and significantly reduces the danger of flares comparing to conventional treatment.^12^ The 3-year follow up from two phase III studies revealed the effectiveness and the high safety profile of administrating Canakinumab to patients with difficult to treat gouty disease.^14^. With the addition of new cases, better conclusions will arise for the use of Canakinumab in gout.
